# Challenge and threat appraisal of job demands and their impact on mental health among vocational education teachers

**DOI:** 10.1186/s12889-026-29796-7

**Published:** 2026-10-08

**Authors:** Nina Marlene Sarochan, Carmela Aprea

**Affiliations:** Business School, Area Economic and Business Education, University of Mannheim, Mannheim, Germany

**Keywords:** Teachers, Cognitive appraisal, Job demands, Well-being, Burnout, Stress, Job satisfaction, Challenge, Threat, Vocational education

## Abstract

**Background:**

Teachers in vocational schools are confronted with many daily job demands. However, these demands are not directly associated with stress. According to the biopsychosocial model of challenge and threat, such demands can be judged as either challenging or threatening. Based on this model, the present study addressed the following research questions: (1) To what extent are different job demands appraised as challenges and threats by teachers in vocational education? (2) How does the appraisal of job demands moderate the relationship between perceived job demands and well-being, job satisfaction, risk of burnout, and experience of stress among teachers in vocational schools? (3) How do the personality traits and resilience of these teachers influence the appraisal of job demands?

**Methods:**

To answer these questions, we drew on data from an online survey conducted in Baden-Württemberg, Germany. With the use of closed items, 1470 teachers in vocational education were asked about their perception of six categories of job demands as well as their personality traits, resilience, tendency to burnout, well-being, job satisfaction, and experience of stress. Descriptive analyses, moderation analyses using structural equation modelling, and path analyses were conducted.

**Results:**

The descriptive analyses showed that the category of *teaching and interacting with learners* was perceived as the most challenging category. Furthermore, challenge appraisal as a moderator was found to increase job satisfaction and decrease levels of burnout, while threat appraisal decreased well-being. Finally, resilience had a significant influence on the appraisal of job demands: higher levels of resilience led to higher values for challenge appraisal and lower values for threat appraisal.

**Conclusion:**

These results imply that the appraisal of job demands is related to the mental health of teachers in vocational education. Building a repertoire of resources, e.g., cognitive appraisal training or cooperation between colleagues, may support a more optimistic challenge appraisal of job demands and improve teachers’ mental health. Furthermore, as resilience has been found to influence this appraisal process in favor of teachers’ mental health, teachers are encouraged to increase their resilience, for example, by using interventions.

**Supplementary Information:**

The online version contains supplementary material available at https://doi.org/10.1186/s12889-026-29796-7.

## Introduction

Teaching is a demanding profession, with numerous job demands shaping teachers’ daily work [1]. As a result, various psychological symptoms are highly prevalent in the teaching profession [2]. Burnout is especially prevalent among a large proportion of teachers [3]. In 2004, Schaarschmidt published a large study revealing that 30% of German teachers show burnout symptoms [4]. A more recent scoping review including studies from around the world found a prevalence of burnout among a quarter of all teachers [5]. Kariou et al. stated that the emotional labor of teachers is especially related to the risk of burnout in this population [6]. Furthermore, burnout rates in teachers have been reported to be higher than in other emotionally demanding jobs, such as medicine, nursing, and social work [2].

The alarming prevalence of burnout among teachers not only impacts their individual well-being but also contributes to broader systemic issues, such as the growing teacher shortage observed in many countries [7, 8]. This shortage poses significant challenges to maintaining the quality and accessibility of education [9], further compounding the pressures on the teaching profession. According to predictions, there will be a shortage of about 25,000 teachers until 2025 in Germany [9]. In vocational schools and vocational subjects within upper-level secondary schools, a shortage of about 5,355 teachers is expected [9]. At the same time, positive teacher well-being is positively associated with central individual and educational outcomes. For example, a review by Dreer reported positive correlations between teacher well-being and teachers’ self-efficacy, quality of instruction, school outcomes, student well-being and student outcomes [10]. Therefore, it is important not only to reduce negative outcomes such as burnout and stress among teachers, but also to promote positive teacher well-being and occupational functioning, especially when aiming to address teacher shortages. However, to do so it is important to understand the appraisal process behind the job demands that teachers face in their daily work. According to Blascovich and Tomaka in their Biopsychosocial Model of Challenge and Threat [11], individuals appraise potentially stressful situations as either challenging or threatening. While a challenge appraisal has been found to lead to positive emotions and mobilization of energy, a threat appraisal is associated with negative emotions and conservation of resources. This can help explain why some teachers thrive in potentially stressful situations and others struggle with them, which might lead to mental health problems and higher drop-out rates.

This paper is structured as follows. We begin by reviewing existing research on job demands in the teaching profession and the process of cognitive appraisal including possible antecedents of this process and outcomes. In the method section, we present our measurement instruments to capture teachers’ perceptions of job demands and the extent to which they see them as challenging and to which as threatening. This section also includes the method of analysis we used. Then, the results are presented and discussed. Finally, the paper concludes with a summary and outlines the implications for teachers and policymakers.

### Job demands in the teaching profession

Teaching has been found to be a particularly demanding job [1, 4], as it requires significant personal (emotional) investment [12]. Thus, some researchers have taken a closer look at the demands made of teachers and categorized them.

Van Dick proposed a model of teachers’ job demands called *Stress Analysis for Teachers*. This model divides job demands into three levels: individual job demands, school-level job demands, and systemic job demands [13]. *Individual job demands* include preparing lessons and follow-up, administrative tasks, disciplinary problems, learners’ lack of motivation, over-involvement of parents/guardians, conflicts with individual colleagues. *School-level job demands* include material school resources, heterogeneous classes, class sizes, noise pollution, and problems with school management. The third level of *systemic job demands* includes unclear work requirements, undefined working hours, conflicts with school authorities, and a lack of public recognition. This model shows that teachers experience demands at different levels [13].

Kyriacou [14] listed similar job demands in the teaching profession and summarized the following as key sources of teachers’ stress: teaching students with a lack of motivation, maintaining discipline, time pressure and workload, dealing with change, being evaluated by others, dealing with colleagues, administration, and management, role conflict and ambiguity, and poor working conditions.

While the models of job demand in the teaching profession by van Dick [13] and Kyriacou [14] have existed for more than 20 years, newer research also looks at teachers’ job demands at different school levels, e.g., vocational education. For instance, Boldrini et al. [15] categorized 24 job demands of vocational education and training (VET) teachers, which differed in part from the job demands of teachers in general education schools. Similar to van Dick [13], Boldrini et al. [15] clustered the challenges into three categories: context-related, individual, and teacher-related challenges. The main differences among the existing models are the additional demands emerging from the dual system of vocational education in Germany. Due to this system, all trainees are employed by a company or training organization alongside their schooling. Additionally, VET teachers have to not only deal with demands from the educational system, but also the labor market and must keep up with changes in their professional field [15]. Therefore, Boldrini et al. [15] identified demands such as *difficult alliances with students’ company trainers* and *pressure from the world of work* as demands specific to this school form. Furthermore, studies have shown that students in VET are more challenging regarding achievement and motivation [15, 16].

These categorizations show that teachers face numerous job demands from various areas of their job. While VET teachers share many job demands of primary and secondary school teachers, there are additional challenges for VET teachers which make transferring research results on teachers in primary or secondary education to VET teachers difficult [15]. Therefore, this article aimed to take a closer look at teachers in VET, their job demands, and how they perceive these demands.

### Cognitive appraisal of job demands

Now that we have addressed the well-researched job demands of the teaching profession, it is important to recognize that dealing with job demands is not directly associated with stress [17]. A model that is often used in research to study the effects of teachers’ job demands on their mental health is the job demands – resources (JD-R) model by Demerouti et al. [18, 19]. The JD-R model distinguishes between job demands and job resources and proposes that well-being and health in the occupational context is a result from a balance between “positive (resources) and negative (demands) job characteristics” [20]. According to this model, high job demands can impair well-being in the occupational context, while job resources can buffer negative outcomes and increase well-being. While the JD – R model explains how job demands and resources influence occupational well-being [21], the present study focuses on how the job demands are evaluated and interpreted as a challenge or threat by individual teachers. Therefore, we draw on the Biopsychosocial Model of Challenge and Threat by Blascovich and Tomaka to take a more detailed look at the appraisal processes of individuals through which job demands are interpreted as challenges or threats [11]. Both models can be considered as complementary in this study. The Biopsychosocial Model of Challenge and Threat [11] explains how individuals appraise potentially stressful situations as either challenging or threatening. This has been a prevailing model in the research on challenge and threat [22] and is based on the research of Lazarus and colleagues on the importance of cognitive appraisal processes in stress and emotion [23]. According to Lazarus and Folkman [17], processes of cognitive appraisal “intervene between the initial perception and subsequent experience of a potentially stressful situation” [23]. They emphasized that even though particular situations and environmental demands produce stress for considerable numbers of people, they still differ in their “sensitivity and vulnerability to certain types of events, as well as in their interpretations and reactions” [17]. Lazarus and Folkman defined cognitive appraisal as “the process of categorizing an encounter, and its various facets, with respect to its significance for well-being” [17] and differentiated between primary and secondary appraisals. Through the primary appraisal, a person evaluates the significance of a situation with regard to their well-being. In secondary appraisal, the person evaluates their available resources for coping and possible courses of action [24]. Building on this research, Blascovich and Tomaka [11] proposed that individuals appraise situations as a threat when it is relevant to the individuals’ goals and that “environmental demands are perceived as taxing or exceeding resources or ability to cope” [23]. In contrast, when the demands are evaluated to be within the individual’s ability to cope or the individual has the necessary resources at their disposal, a challenge appraisal occurs [23]. Thus, the Biopsychosocial Model of Challenge and Threat includes two appraisal components: “primary appraisals of situational demands and secondary appraisals of coping abilities” [25].

While this model has been widely applied to domains such as sports and motivated behavior [22], only a few studies have addressed the relationship between the cognitive appraisal of job demands and teacher (mental) health and well-being [2, 26]. The present study aimed to address this research gap and elucidate the underlying mechanisms of the cognitive appraisal of teachers, especially in vocational education. In addition to the process of appraisal, the possible antecedents and outcomes of this process are considered.

#### Antecedents of the appraisal process

The model of challenge and threat has often been tested with regard to acute reactions to particular stressors. Moreover, challenge and threat appraisals are defined as perceived balance between situational demands and resources [11] which shows that appraisal is a situation-dependent process. However, “it has been maintained in the model that individuals have tendencies to consistently appraise events as challenging or threatening” [25]. These considerations are also in line with more recent developments in the JD-R model, where the role of personal resources in explaining employees’ mental health is emphasized. In the JD-R model, personal resources are “positive self-evaluations that refer to individuals’ sense of their ability to control and impact their environment successfully” [27, 28]. This suggests that the consequences of job demands may not only depend on the job demand itself but also on the individual and its resources. While several studies have linked personality to appraisal processes, only a few have used the Biopsychosocial Model of Challenge and Threat [11] as a guiding framework. In these studies, personality dimension of neuroticism (N) was found to predict stress-related negative emotions, while appraisals mediated this relationship [25, 29, 30]. Furthermore, extraversion (E) and openness (O) predicted high positive emotion, while appraisals partially mediated the effect of openness [25, 30]. Tomaka and Magoc summarized the state of the literature on the relationship between personality and challenge or threat appraisals as incomplete [25]. They stated that while neuroticism, extraversion, and, to a lesser extent, openness correlate consistently with measures of appraisals, results regarding the other two dimensions of the Big Five personality traits, agreeableness (A) and conscientiousness (C), are less consistent [25].

In addition to personality, it has been suggested that resilience increases challenge appraisals [31]. Resilience is often conceptualized as the capacity for positive adaptation in the context of adversity [32] and thus might serve as an indicator of the individuals’ broader capacity to cope with difficult situations. Furthermore, in the context of the teaching profession, resilient teachers have been found to be able to thrive in difficult situations, focusing on the positive, experience increased commitment to the teaching profession [33, 34], and to be better equipped to navigate the demands in their daily work [35]. Therefore, resilient teachers might be more likely to perceive job demands as manageable and appraise them more as a challenge. In addition, threat appraisals are a sign of vulnerability [31]. Seery [31] found that low-to-moderate levels of adversity contribute to building resilience, which in turn makes future adverse events, such as job demands, seem more manageable. These findings are in line with the concept of psychological toughness developed by Dienstbier [36], which is a foundational concept of the Biopsychosocial Model of Challenge and Threat [25]. Considering the JD-R model and its more recent developments, personal resources are increasingly recognized as a relevant aspect in explaining how individuals respond to job demands. More specifically, it has been proposed that personal resources, like resilience and self-efficacy, are reciprocally related to job resources. Thus, individuals with more personal resources are more likely to have access to more job resources, and the other way around [27]. According to the JD-R theory, resilience may therefore be a personal resource that helps understand individual differences in appraising job demands. However, evidence on the effects of resilience on the appraisal process and the mechanisms behind it are scarce.

#### Outcomes of the appraisal process

Lazarus and Folkman [17] stated that dealing with potentially stressful situations does not necessarily lead to feelings of stress for everyone, as it is dependent on the individual appraisal of the situation at hand. The outcome of the appraisal process has important emotional and behavioral consequences [11, 25]. Based on the Biopsychosocial Model of Challenge and Threat [11], a challenge appraisal leads to positive affect or low negative affect, mobilizes energy, and induces anticipation of success. A threat appraisal is characterized by high negative affect, conservation of resources, and anticipation of failure [23, 25]. This has been found to increase feelings of stress [17]. The aforementioned relationship has been found in many domains, such as health care [37] and sports [22], as well as in the educational context [e.g., 1]. In their study on the relationship between cognitive appraisal and psychological distress in schoolteachers, Seixas et al. [1] found that threat perception was a significant predictor of teachers’ mental health and feelings of distress. However, challenge perception was not found to predict teachers’ mental health. In addition, it has been shown that occupational stress leads to a decrease in job satisfaction [1, 38, 39]. Specifically, the impact of stress on teachers’ well-being and burnout has been extensively researched [1]. For example, Gomes and colleagues investigated the effect of cognitive appraisal on the relationship between job demands and burnout for academic teaching staff [26]. They found that primary cognitive appraisal was positively associated with burnout when perceiving a situation as a threat but negatively associated when viewing it as a challenge. Furthermore, Troesch and Bauer [40] emphasized that while dealing with teaching-related demands can lead to feelings of stress, they can also be important sources of professional development. This shows that challenge appraisals can promote positive occupational functioning and are not only associated with the absence of negative outcomes. Challenges imply the potential of personal growth and promotion of mastery and employees perceive demands that are appraised as challenges as chances to learn and achieve [41]. Crawford and colleagues found that challenging demands were positively related to engagement in employees [41].

It is important to mention that challenge and threat appraisals are not considered as opposing ends of a continuum. Lazarus and Folkman state that both “can occur simultaneously, and must be considered as separate, although often related, constructs” [17]. Furthermore, it should be recognized that appraisals are, as mentioned above, dependent on the specific situation and availability of resources in the specific situation [42]. Therefore, appraisals for different job demands are not considered fixed and have been shown to have in-person variability throughout the day [43, 44].

### Research questions

Based on the research results outlined in the previous section, this study addressed the following research questions:

- Research Question 1 (RQ1): To what extent are different job demands appraised as challenges and threats by teachers in vocational education?
- Research Question 2 (RQ2): How does the appraisal of job demands moderate the relationship between perceived job demands and well-being, job satisfaction, risk of burnout, and experience of stress among teachers in vocational schools?

With regard to the second research question, the following two hypotheses, H1 and H2, were derived using the Biopsychosocial Model of Challenge and Threat [11]:

- H1: With a challenge appraisal, teachers have lower experiences of stress and risk of burnout, and higher well-being and job satisfaction.
- H2: With a threat appraisal, teachers have higher experiences of stress and risk of burnout, and lower well-being and job satisfaction.

- Research Question (RQ3): How do the personality and resilience of these teachers influence the appraisal of job demands?

Based on RQ3, hypothesis H3 was formulated:

- H3: Teachers with higher levels of resilience are more likely to appraise job demands as a challenge and less likely to appraise them as a threat.

## Methods

### Participants and data collection

This study uses the data of the German project called *Working Hours*,* Workload*,* and Resilience of Vocational Education Teachers in Baden-Württemberg*,* Germany* (German acronym AARL-BS, short for Arbeitszeit, Arbeitsbelastung und Resilienz von Lehrkräften an beruflichen Schulen in Baden-Württemberg) by the Vocational Education Teachers’ Association of Baden-Württemberg, Germany (BLV BW e.V.) and the University of Mannheim. This cross-sectional data collection was carried out between February and November 2022, using an online self-report questionnaire administered via the SoSci Survey tool. The data collection resulted in a database of 1470 vocational education teachers without a management function; this group is the basis for the further data analysis in this study. The sample included 56.9% females and was, on average, 44.89 years old (*SD* = 9.97). 60.3% of the participating teachers worked full-time, 19.7% worked part-time at 75–99%, 16.6% worked 50–75%, and 3.6% worked less than 50%. While this sample is representative for the federal state Baden-Württemberg, Germany regarding the demographic variables gender, age, job percentage and regional council, the timing of data collection might be a limiting factor of overall representativeness. As data was collected in 2022 when school life was still heavily affected by the Covid-19 pandemic and the associated restrictions. Participation in the study was voluntary. After signing up, all participants received information on the study’s objectives, data confidentiality, and terms of data use. Electronic informed consent was obtained prior to accessing the online questionnaire. The full survey used in this study can be found in the supplementary files.

### Measures

The job demands, their appraisal and personality traits, and the measures of teachers’ work experience—well-being, job satisfaction, risk of burnout, and experience of stress—were collected via online self-report questionnaires, delivered via the online survey tool called SoSci Survey.

To measure the job demands of teachers in vocational education, a questionnaire called *Assessment of Demanding Aspects for Vocational Education Teachers* (ADA-VET) was used. It originated in the study by Boldrini et al. [15] in Switzerland and was adapted to the German context by the authors. The questionnaire consists of 21 items assessing six categories: (1) job demands in the context of the social and educational environment, (2) social job demands within the teaching staff, (3) social job demands with the school management, (4) job demands in the context of task scope and complexity, (5) job demands in the context of teaching or interacting with learners, and (6) job demands in the context of the school framework. The items represent different demands that teachers can rate in terms of frequency of occurrence on a 5-point Likert scale: 1 = never, 2 = every few months, 3 = every few weeks, 4 = every few days, and 5 = daily. The full questionnaire can be found in Appendix (1) The six-factor structure of the job demands was tested using a confirmatory factor analysis. The model showed an acceptable overall fit to the data, χ²(174) = 1177.00, *p* < .001, CFI = 0.903, RMSEA = 0.067, and SRMR = 0.068. Standardized factor loadings ranged from 0.32 to 0.87, with 20 of the 21 items showing factor loadings of ≥ 0.43. A table with standardized factor loadings for all 21 items is provided in Appendix (2) Overall, the results provide empirical support for the proposed six-factor structure.

To operationalize the appraisal of these job demands, challenge and threat were assessed as separate dimensions as this is in line with the conceptualization that they are not mutually exclusive and can appear at the same time [17]. The participants were asked to indicate the extent to which they perceived each job demand as a challenge and to which extent they perceived them as a threat. For both scales this was answered on a 4-point Likert scale ranging from 1 = does not apply at all to 4 = applies completely. For perceived frequency of job demands, their challenge appraisal and threat appraisal, we used the six subdimension of the questionnaire. The correlations among the six subdimensions for each measurement variant are presented in Appendix 3.

To assess the effect of teachers’ appraisals of job demands on their working experience, measures for well-being, job satisfaction, risk of burnout, and experience of stress were collected.

The World Health Organization – Five Well-Being Index (WHO-5) was used in its German version to measure teachers’ the overall well-being [45, 46] (see Appendix 4). This standardized scale consists of five items (e.g., *I feel cheerful and in good spirits*) on a 5-point Likert scale (1 = *at no time* to 5 = *all of the time*). While this measure is not specific to the professional context, it serves as a widely validated and established screening tool for overall well-being [47].

*Job satisfaction* was measured with the following item: *Considering everything that plays a role in your work as a teacher at vocational schools: How satisfied are you overall with your professional situation at the moment?* The participating teachers could answer this item on a 5-point Likert scale (1 = very unsatisfied to 5 = very satisfied).

To assess teachers’ work-related burnout, the *Burnout Assessment Tool (BAT)* by Schaufeli et al. [48] was used in its German version [49] (see Appendix 5). This standardized tool consists of 12 statements (e.g., *At work*,* I feel mentally exhausted*) divided into four subscales: exhaustion, mental distance, cognitive impairment, and emotional impairment. All items were rated on a 5-point Likert scale (1 = *never* to 5 = *daily*). This scale was used because it has been validated for the occupational context [48] as well as for teachers [50] and applied in research on teachers and other occupational groups which facilitates comparability.

*Experience of stress* was measured with a single item: *Considering everything that plays a role in your work as a teacher at vocational schools: How often do you feel exposed to situations that you experience as stressful?* The participating teachers could answer this item on a 5-point Likert scale (1 = never, 2 = every few months, 3 = every few weeks, 4 = every few days, 5 = every day).

Regarding the antecedents in the model, the personality of teachers at vocational schools was assessed using the German version of the 10-item Big Five Inventory (BFI-10) [51] (see Appendix 6). This scale is well-established and psychometrically validated in its German as well as English version [52]. It consists of ten items, two for each of the five personality dimensions (openness, conscientiousness, extraversion, agreeableness, and neuroticism), e.g., “I see myself as someone who is reserved,” “I see myself as someone who is generally trusting,” and “I see myself as someone who is outgoing, sociable.” These items were assessed on a 5-point Likert scale ranging from 1 = *disagree strongly* to 5 = *agree strongly*.

To assess the resilience of the participating teachers, the German version of the Brief Resilience Scale (BRS) by Smith et al. [53, 54] was applied (see Appendix 7). This scale consists of six items (e.g., *I tend to bounce back quickly after hard times*; *I have a hard time making it through stressful events*) with a 5-point Likert scale (1 = *strongly disagree* to 5 = *strongly agree*).

### Data analysis

As all items in the final questionnaire had a closed response format, the means of the following items were calculated at the participant level for the Brief Resilience Scale, job satisfaction, experience of stress, and the Burnout Assessment Tool. For the Big Five Inventory, mean values were calculated for the five subscales of openness, conscientiousness, extraversion, agreeableness, and neuroticism. For the WHO-5 scale, a summative score was calculated instead of the mean, in accordance with the questionnaire manual [45].

To answer the first research question, descriptive analyses of job demands and their appraisal were conducted for the subcategories of job demands.

To answer the second research question, two path analyses were carried out in jamovi (Version 2.7.39) using the SEMj module for both challenge appraisal and threat appraisal as the moderators. For job demands, challenge and threat appraisal, we used the six subcategories due to complexity reasons. The six categories of perceived job demands were included as antecedents, challenge or threat appraisal for the six categories were included as moderators, and all four mental health outcomes (well-being, job satisfaction, risk of burnout, and experience of stress) were included in the model as outcomes. Each model included six specified antecedent x moderator interactions, with each appraisal category moderating the association between its corresponding job demand category and the four outcomes. Antecedents, moderators and residuals of the outcomes were allowed to correlate.

For the third research question, two path analyses were conducted in jamovi (Version 2.7.39) using the SEMj module. In the first model, challenge appraisal for the six categories of job demands were entered as simultaneous outcomes. In the second model, threat appraisal for the six categories of job demands were used as simultaneous outcomes. Resilience and the five personality dimensions of the Big Five Inventory (openness, conscientiousness, extraversion, agreeableness, and neuroticism) were used as antecedents in both models. To account for the expected associations among individual characteristics, all predictors were allowed to covary. In addition, residual covariances among the six appraisal categories were freely estimated, reflecting the assumption that appraisals of different categories of job demands may be interrelated beyond the effects of resilience and personality traits.

For all analyses, effect sizes were interpreted according to Ellis’s convention [55]. For moderation analyses and path analyses, *R*^*2*^ was used as the effect size for the model overall and was interpreted as a small effect for *R*^*2*^ from 0.02 to 0.13, a medium effect for 0.13 to 0.26, and as a large effect for *R*^*2*^ over 0.26. Additionally, standardized β were used as the effect size for the individual variables.

## Results

### Research question 1: perception of job demands in VET

To answer the first research question (To what extent are different job demands appraised as challenges and threats by teachers in vocational education? ), descriptive analyses of the six categories of job demands and their appraisal were conducted (see Table 1). The most frequently perceived subcategory was the category of job demands in the context of *task scope and complexity* (*M* = 3.96, *SD* = 0.74), closely followed by the category of job demands in the context of *teaching and interacting with learners* (*M* = 3.94, *SD* = 0.79). Descriptive analyses of the appraisal of job demands showed that the category of job demands with the highest scores in challenge appraisal is the category of *teaching and interacting with learners*. The category with the highest values in threat appraisal is the category *school management*.

**Table 1:** Descriptive analysis of perceived job demands and their appraisal

	Perception of Job Demands		Challenge Appraisal of Job Demands		Threat Appraisal of Job Demands	
Category of job demands	*n*	M(SD)	*n*	M(SD)	*n*	M(SD)
1 – Social and educational environment	1275	2.89 (0.81)	890	2.17 (0.70)	898	2.42 (0.69)
2 – teaching staff	1274	2.32 (0.86)	608	1.88 (0.64)	635	2.61 (0.74)
3 – school management	1273	2.27 (0.97)	592	1.64 (0.67)	610	3.06 (0.76)
4 – task scope and complexity	1276	3.96 (0.74)	1047	1.96 (0.67)	1075	2.96 (0.70)
5 – teaching and interacting with learners	1276	3.94 (0.79)	1011	2.38 (0.67)	1027	2.69 (0.68)
6 – school framework	1276	3.31 (0.87)	878	1.74 (0.63)	914	2.90 (0.68)

*n*  sample size, *M*  mean, *SD*  standard deviation

### Research question 2: effect of appraisal on the mental health of VET teachers

To answer the second research question, moderation analyses using structural equation modeling were conducted. It was investigated whether the relationship between the six categories of job demands (antecedents) and the mental health of teachers, particularly well-being, job satisfaction, risk of burnout, and experience of stress (outcomes) are influenced by the appraisal of the job demands (moderator). Two separate models were calculated: one with challenge appraisal as the moderator and one with threat appraisal as the moderator.

The results of the model using challenge appraisal for the six categories of job demands as a moderator showed several direct associations between perceived job demands and the mental health-related outcomes. For well-being, the categories of *school management* (β= -0.259, *p* = .017) and *task scope and complexity* (β= -0.238, *p* = .028) were negatively associated. The category of *school management* was also negatively associated with job satisfaction (β = -0.411, *p* < .001). Thus, more frequent perception of these job demands was related to lower well-being and job satisfaction in teachers. Regarding risk of burnout, the category *social and educational environment* (β = 0.263, *p* = .008), teaching staff (β = 0.225, *p* = .043), and *task scope and complexity* (β = 0.240, *p* = .017) had a positive relationship. For experience of stress, the category *task scope and complexity* (β = 0.328, *p* = .001) was positively associated. This means that more frequently perceived job demands in these categories are related to higher risk of burnout and experience of stress.

Regarding the interaction terms between perceived job demands and challenge appraisal, two interaction effects reached statistical significance. The relationship between perceived job demands in the category of *social and educational environment* and risk of burnout was significantly moderated by challenge appraisal (β = -0.307, *p* = .046). The interaction effect was negative, indicating that higher challenge appraisal in this category weakened the positive association between the perception of the corresponding job demands and risk of burnout. Furthermore, the relationship between perceived job demands in the category of *school management* and job satisfaction was significantly moderated by challenge appraisal in this category (β = 0.374, *p* = .020). As this interaction effect is positive, it suggests that higher challenge appraisal reduces the negative association between these job demands and job satisfaction.

In the model with threat appraisal as the moderator, several significant direct associations were found between perceived job demands and the outcome of experience of stress. The category *social and educational environment* (β = 0.284, *p* = .013), and *task scope and complexity* (β = 0.332, *p* = .012) were positively associated with experience of stress, indicating that more frequently perceived job demands was related to higher perceived stress. For well-being, the category *school management* showed a negative association with WHO-5 scores on significance level of *α* = 0.10 (β = -0.384, *p* = .056), suggesting that more frequently perceived job demands in this category are related to lower well-being. Regarding the interaction terms for threat as the moderator, one interaction effect reached statistical significance. The interaction between category of *task scope and complexity* and threat appraisal in this category was significantly related to well-being (β = -0.595, *p* = .042). This negative interaction suggests that higher threat appraisal in this category increases the negative association between job demands and well-being in this category.

### Research question 3: influence of resilience and personality traits on the appraisal of job demands

To answer the question of how the resilience and Big Five Personality traits of teachers in vocational education influence the appraisal of job demands, two path analyses were carried out: one for challenge appraisal and one for threat appraisal. Table 2 includes an overview of all *R*^*2*^ values for challenge and threat appraisal in each category and Table 3 includes an overview of all parameters estimates in the path analyses.

**Table 2:** Explained variances for challenge and threat appraisal

	Category 1	Category 2	Category 3	Category 4	Category 5	Category 6
*R*^2^ for challenge appraisal	0.046	0.028	0.020	0.067	0.043	0.018
R^2^ for threat appraisal	0.057	0.052	0.029	0.086	0.032	0.030

*R*^*2*^explained variance

**Table 3:** Path analyses results for the association between personality traits and resilience and appraisal

		Challenge Appraisal			Threat Appraisal		
	Antecedent	B (SE)	β	p	B (SE)	β	p
Category 1 - social and educational environment	BFI – O	0.003 (0.024)	0.004	0.897	0.021 (0.023)	0.029	0.366
	BFI – C	0.116 (0.031)	0.124	< 0.001	-0.004 (0.031)	-0.004	0.893
	BFI – E	-0.023 (0.026)	-0.029	0.390	0.029 (0.026)	0.037	0.263
	BFI – A	0.098 (0.033)	0.100	0.003	-0.081 (0.032)	-0.083	0.012
	BFI - N	-0.006 (0.034)	-0.007	0.851	0.075 (0.034)	0.086	0.025
	BRS	0.124 (0.037)	0.126	< 0.001	-0.158 (0.037)	-0.162	< 0.001
Category 2 - teaching staff	BFI – O	0.040 (0.026)	0.060	0.123	-0.012 (0.030)	-0.016	0.685
	BFI – C	0.108 (0.034)	0.127	0.001	0.0282 (0.040)	0.028	0.482
	BFI – E	-0.012 (0.029)	-0.017	0.670	0.003 (0.034)	0.004	0.927
	BFI – A	-0.031 (0.036)	-0.035	0.379	-0.006 (0.042)	-0.006	0.888
	BFI - N	-0.065 (0.036)	-0.081	0.071	0.078 (0.042)	0.083	0.064
	BRS	0.001 (0.039)	0.001	0.973	-0.182 (0.046)	-0.174	< 0.001
Category 3 – school management	BFI – O	0.038 (0.027)	0.054	0.153	0.005 (0.032)	0.007	0.869
	BFI – C	0.062 (0.035)	0.070	0.071	0.029 (0.041)	0.028	0.488
	BFI – E	-0.00451 0.0297	-0.006	0.879	0.068 (0.036)	0.080	0.055
	BFI – A	0.05691 0.0366	0.060	0.120	-0.153 (0.044)	-0.142	< 0.001
	BFI - N	0.01964 0.0382	0.023	0.607	-0.060 (0.045)	-0.062	0.183
	BRS	0.085 (0.042)	0.090	0.042	-0.091 (0.050)	-0.085	0.065
Category 4 – task scope and complexity	BFI – O	-0.016 (0.022)	-0.023	0.450	0.054 (0.020)	0.079	0.007
	BFI – C	0.116 (0.028)	0.127	< 0.001	-0.021 (0.026)	-0.024	0.422
	BFI – E	-0.027 (0.024)	-0.036	0.245	0.036 (0.022)	0.049	0.103
	BFI – A	0.065 (0.030)	0.067	0.029	-0.018 (0.028)	-0.020	0.511
	BFI - N	-0.014 (0.030)	-0.016	0.650	0.063 (0.028)	0.077	0.025
	BRS	0.192 (0.033)	0.201	< 0.001	-0.212 (0.031)	-0.233	< 0.001
Category 5 – teaching and interacting with learners	BFI – O	0.044 (0.022)	0.063	0.044	-0.017 (0.023)	-0.023	0.451
	BFI – C	0.079 (0.028)	0.088	0.005	0.012 (0.029)	0.012	0.688
	BFI – E	0.004 (0.024)	0.005	0.878	-0.009 (0.025)	-0.012	0.712
	BFI – A	0.072 (0.030)	0.076	0.016	-0.061 (0.031)	-0.061	0.050
	BFI - N	-0.028 (0.031)	-0.033	0.371	0.069 (0.033)	0.077	0.035
	BRS	0.116 (0.034)	0.123	< 0.001	-0.104 (0.036)	-0.105	0.004
Category 6 – school framework	BFI – O	-0.024 (0.022)	-0.036	0.272	0.033 (0.023)	0.047	0.152
	BFI – C	0.082 (0.028)	0.096	0.004	0.075 (0.030)	0.082	0.013
	BFI – E	-0.008 (0.025)	-0.011	0.759	0.004 (0.026)	0.005	0.880
	BFI – A	0.044 (0.030)	0.049	0.138	-0.025 (0.032)	-0.025	0.435
	BFI - N	-0.013 (0.031)	-0.016	0.689	0.029 (0.033)	0.033	0.383
	BRS	0.049 (0.034)	0.055	0.154	-0.117 (0.036)	-0.122	0.001

*BRS*  Measure of resilience, *BFI*  Big Five Inventory, *O*  openness, *C*  conscientiousness, *E*  extraversion, *A*  agreeableness, *N*  neuroticism, *B*  unstandardized regression coefficient, *SE*  standard error, *β*  standardized regression coefficient, *p*  p-value

The model with challenge appraisal as the outcome explained a small proportion of variance in challenge appraisal of the job demand categories. Explained variances are between *R*^*2*^ = 0.018 for category *school framework* and *R*^*2*^ = 0.067 for category *task scope and complexity*. Resilience was significantly positively related to challenge appraisal for several categories. The strongest association was observed for appraisal of category *task scope and complexity* (β = 0.201, *p* < .001). Further significant associations were found for category *social and educational environment* (β = 0.126, *p* < .001), *school management* (β = 0.090, *p* = .042), and *teaching and interacting with learners* (β = 0.123, *p* < .001). For personality traits of teachers, conscientiousness was significantly positively associated with challenge appraisal of five categories, with the strongest association for the categories *teaching staff* and *task scope and complexity* (β = 0.127, *p* = .001 for both). Agreeableness was positively associated with challenge appraisal of categories *social and educational environment* (β = 0.100, *p* = .003), *task scope and complexity* (β = 0.067, *p* = .029), and *teaching and interacting with learners* (β = 0.076, *p* = .016). Openness was significantly positively associated with challenge appraisal for category teaching and interacting with learners (β = 0.063, *p* = .044). No significant associations were found for extraversion and neuroticism.

For the model for threat appraisal, the explained variances were also small ranging from *R²* = 0.029 for category *school management* to *R²* = 0.086 for category *task scope and complexity*. Resilience was found to be significantly negatively associated with threat appraisal for five out of the six categories. The strongest relation was with the category *task scope and complexity* (β = − 0.233, *p* < .001). Neuroticism was positively associated with threat appraisal for categories *social and educational environment* (β = 0.086, *p* = .025), *task scope and complexity* (β = 0.077, *p* = .025), and *teaching and interacting with learner*s (β = 0.077, *p* = .035). Agreeableness was negatively associated with threat appraisal for the categories *social and educational environment* (β = − 0.083, *p* = .012), and *school management* (β = − 0.142, *p* < .001). For openness and conscientiousness only one significant relation was found. Openness was positively related to threat appraisal in category *task scope and complexity* (β = 0.079, *p* = .007). Conscientiousness was positively associated with threat appraisal in category *school framework* (β = 0.082, *p* = .013).

## Discussion

The aim of this study was to gain a deeper understanding of how teachers in vocational education perceive job demands in their day-to-day working lives. The focus was on the subjective appraisal of these job demands, the influence on teachers’ mental health, and the possible influence of personality traits and resilience on the appraisal process. We addressed three central research questions.

### Research question 1

Regarding research question 1 on teachers’ perception of their job demands, the categories of *task scope and complexity* and *teaching and interacting with learners* had the highest mean values, suggesting that these demands were perceived most frequently in the sample of teachers in vocational education. However, the category *teaching and interacting with learners* was also perceived as most challenging. One possible explanation for this comparatively high value in challenge appraisal in the category of *teaching and interacting with learners* is that these activities present a core aspect of the teaching profession. In addition, teacher education is designed to prepare teachers to be able to deal with different student-related demands. Therefore, teachers might have more resources available for dealing with these types of job demands. Based on the JD-R model, this can make job demands seem more manageable and buffer the negative effects of these demands [21, 18]. Furthermore, research has shown that the teachers’ relationship to their students is of central importance for their motivation and engagement [56]. Thus, job demands of the category *teaching and interacting with learners* might be among the more intrinsically rewarding aspects of the job. The category that was perceived as most threatening was *school management*, closely followed by *task scope and complexity*. This shows that the mere consideration of how frequently job demands are perceived is insufficient as a basis for interpretation. Overall, the mean values for threat appraisal were found to be higher than those of challenge appraisal. This suggests that the participating teachers in vocational education perceived the presented job demands as more threatening than challenging. In line with the considerations of the appraisal of job demands, the term *challenging* refers to a job demand that is feasible or manageable with the available resources. Threatening, on the other hand, refers to a job demand that exceeds an individual’s ability to cope or the available resources [23]. However, it should be noted that the values of challenge and threat appraisal were very close to each other and often ranged around the middle of the scale. This shows that the differentiation between challenge and threat might not be as distinct. Furthermore, it should be considered that the appraisal of job demands is a situation-dependent process, and several diary studies have found in-person fluctuations of appraisal throughout the day [43, 44]. Therefore, job demands cannot be described as inherently challenging or threatening. These results represent more of a situational assessment of the appraisal of teachers in vocational education. 

### Research question 2

Research question 2 and the corresponding hypotheses (H1 and H2) focused on the effects of the appraisal on the four outcomes related to mental health: well-being, job satisfaction, risk of burnout, and experience of stress. Two moderation analyses using structural equation modelling were conducted with the six categories of job demands as antecedents, challenge and threat appraisal as moderators, and mental health-related measures as outcomes. Two moderation effects were found for the model using challenge appraisal as the moderator. Challenge appraisal was found to moderate the association between the perceived job demands in category *school management* and job satisfaction. The relation between these job demands and job satisfaction was negative. The positive interaction of challenge appraisal as the moderator indicates that challenge appraisal weakened the negative association between job demands in the category *school management* and job satisfaction. The other significant moderation effect was found for the negatively connoted outcome risk of burnout and the category of job demands *social and educational environment*. The negative interaction of challenge appraisal suggests that when challenge appraisal for these job demands is higher, the positive association between the perception of these demands and risk of burnout is weakened. This shows that when job demands are interpreted more as challenges, they seem more manageable and might therefore be less detrimental for job satisfaction and burnout, even when these job demands are experienced frequently. For H1 we hypothesized that with a challenge appraisal, teachers have lower experience of stress and risk of burnout, and higher well-being and job satisfaction. Our findings, however, only partially support this hypothesis. While results for job satisfaction and risk of burnout are in the expected direction, no significant moderating effects were observed for well-being and experience of stress. Therefore, H1 was only partially confirmed.

Regarding threat appraisal, one significant moderation effect was observed. Threat appraisal was found to moderate the association between job demands in the category of *task scope and complexity* and well-being. The negative association between job demands in this category and well-being was stronger for higher values of threat appraisal in these demands. For H2 we hypothesized that with a threat appraisal, teachers have higher experience of stress and risk of burnout, and lower well-being and job satisfaction. While proof for this hypothesis was only found for well-being, it was in the expected direction as higher threat appraisal was related to lower well-being.

Overall, the findings provide only limited support for the moderating role of cognitive appraisal in the relationship between perceived job demands and mental health-related outcomes. However, the findings are in line with the Biopsychosocial Model of Challenge and Threat indicating that challenge appraisals are related to positive affect and low negative affect, whereas threat appraisals are related to high negative affect [11]. 

### Research question 3

To examine whether teachers’ resilience and personality traits have an influence on the appraisal of job demands (RQ3), two path analyses were conducted. Overall, the findings suggest that resilience has the most consistent associations with appraisal tendencies, whereas the Big Five personality traits show more selective associations with appraisal of some categories.

A central finding was that resilience was positively related to challenge appraisal in four out of six categories of job demands and negatively related to five out of six categories. This indicates that higher resilience was associated with higher values in challenge appraisal and lower values in threat appraisal. These results suggest that resilience might be related to appraisal processes generally and is not limited to the appraisal of individual job demands. As more resilient teachers have been found to be better equipped to navigate the demands in their daily work [35], they might perceive demanding situations as challenges that can be mastered, which then leads to higher challenge appraisal and lower threat appraisal. These findings are also in line with the more recent developments of the JD-R model of personal resources explaining individual differences in responses to job demands [27]. In the context of teachers in VET, resilience may therefore be an important personal resource in interpreting job demands that supports challenge appraisal and reduces threat appraisal. However, this remains subject to further research as the cross-sectional design of this data collection limits the ability to make statements about causal relationships between variables. In H3 we hypothesized that teachers with higher resilience are more likely to appraise job demands as a challenge and less likely to appraise them as a threat. Although resilience was not significantly related to challenge and threat appraisal for all categories of job demands, overall the findings provide broad support for the hypothesis.

Regarding the Big Five personality traits, conscientiousness was found to be an important antecedent for challenge appraisal, showing positive associations for five of the six categories. This suggests that teachers with higher conscientiousness tend to appraise job demands higher on the challenge scale. Conscientiousness “characterizes people who are hardworking, purposeful, and disciplined rather than laid-back, unambitious, and weak-willed” [57]. This description relates to the results presented above: the more hardworking and purposeful a teacher is, the more likely they are to be willing to perceive the demanding aspects of the profession as challenges and addressing them. However, in the model for threat appraisal, consciousness only showed significant negative associations with one category. Agreeableness was positively related to challenge appraisal in three categories and negatively related to threat appraisal in two categories. This indicates that teachers with higher agreeableness were more likely to appraise job demands as challenges and less likely to appraise them as threats. Agreeableness “contrasts generosity, honesty, and modesty with selfishness, aggression, and arrogance” [57], which is in line with these findings. The personality trait of neuroticism showed a clear pattern of positive association with threat appraisal in several categories but was not significantly related to challenge appraisal. The positive relation to threat appraisal suggests that teachers with higher neuroticism tend to appraise job demands higher on the threat appraisal scale. The high pole of neuroticism is characterized by traits such as “sad and scared,” while the “opposing pole is defined by traits such as calm and stable” [57]. Regarding this definition of neuroticism, our findings can be explained as follows: Teachers with high levels of neuroticism may be more scared and emotionally unstable and therefore perceive job demands in these categories as more threatening. Openness and extraversion did not show a lot of significant relations to either challenge or threat appraisal. While openness was positively related to both challenge and threat appraisal for one category, extraversion was not found to be significantly related to any category. The personality dimension of openness to experiences “describes imaginative, curious, and exploratory tendencies as opposed to rigid, practical, and traditional tendencies” [57]. Given this definition of openness, the result of openness being positively associated to threat appraisal in one category is surprising and further research to explain this relationship is necessary.

While generally the effects were small, the pattern of results indicates that differences in resilience and personality traits in teachers are related to how they appraise situations in the work context. However, it is important to mention that while the appraisal process might be partially influenced by individual characteristics like personality traits and resilience, these aspects do not determine the appraisal of a demanding situation independently of the situational context. This follows from the definition of appraisal in the Biopsychosocial Model of Challenge and Threat [11] itself but also emerges from research on the effect of personal vs. situation characteristics on appraisal [58].

### Strengths and limitations

To our knowledge, this study presents the most extensive data collection of job demands and their appraisal among VET teachers in Germany. Even though the large sample size is a major strength of the study and allows for many different analyses, the findings cannot be generalized. Data were collected in only one federal state of Germany, and due to voluntary participation, a self-selection bias might be at play. Further limitations regarding generalizability arise from the specific timing of the survey. The survey was carried out in 2022 at the end of the COVID-19 pandemic. As this study centers on the individual perceptions of VET teachers and their job demands, this perception might be particularly influenced by the circumstances of school closures and online teaching. A study from the same year showed that well-being among the general population was lower than usual [59], which might also affect teachers’ perceptions. Therefore, the broader societal context in Germany at that time must be considered, especially the situation in schools at the time. A follow-up study with greater distance from the pandemic could help with the issue of generalizability.

Furthermore, the unique role of vocational education within the German educational system must be considered. As previously mentioned, scientific insight in this area remains limited, and this study contributes to the existing body of research by focusing on a largely understudied group of teachers.

A further limitation of the study lies in its exclusive reliance on self-reported data from a single cross-sectional survey. The cross-sectional data collection also resulted in a lack of causality. Thus, with this type of data, it cannot be ruled out, e.g., that the outcome variables also influenced the appraisal of job demands. Therefore, further research is needed to identify causal relationships. Furthermore, as all constructs were assessed using self-report scales, it might increase the risk of common method bias and inflate associations between variables [60]. A further limitation concerns the assessment of job satisfaction and experience of stress, which were measured using single-item indicators and therefore provide a less differentiated assessment than multi-item scales. Therefore, findings related to these measures should be interpreted with caution. Regarding the measurement of teachers’ job demands, the present study assessed the perceived frequency of these demands rather than their subjective intensity. Future research should therefore differentiate between the perceived frequency, subjective intensity, and stressfulness of job demands to gain a more detailed understanding of the relationship between job demands and teachers’ mental health.

### Implications and further research

The results of this study indicate that the appraisal of job demands in the daily working lives of teachers in vocational education is related to their mental health and highlight the potential relevance of teachers’ cognitive appraisal processes for understanding differences in how vocational education teachers experience and respond to job demands in their daily working lives. The findings of this study suggest that appraising job demands as challenges rather than threats was associated with more favorable levels of vocational education teachers’ well-being, job satisfaction, and burnout. Therefore, it seems plausible to support VET teachers in gaining the perspective of a challenge appraisal. One potential way of doing this may be by implementing interventions targeting teachers’ cognitive appraisal processes. For example, stress appraisal training or cognitive reframing strategies could help teachers change their perspective from job demands being rather threatening to a more challenge-oriented appraisal. At the same time, interventions aimed at strengthening the resilience of teachers may also indirectly support mental health, as resilience was found to influence the appraisal process of teachers. A study by Kangas-Dick and O’Shaughnessy showed that that the resilience of teachers can be increased by means of resilience interventions and training programs [61]. While these approaches may represent promising practical implications, their effects were not directly examined in the present study. Further empirical research is therefore needed to determine whether, and to what extent, they influence teachers’ appraisal processes and mental health.

In addition to these teacher trainings, changes to the work structure to improve the work experience and mental health in this profession could also help. One possible course of action could be the provision of support services by policy makers or school management, particularly for educators who perceive these demands as threatening. Furthermore, opportunities for collaboration among colleagues might help in dealing with educational job demands. The resources available are an important part of the secondary appraisal process [17, 24]. Individuals appraise job demands as a threat when they do not have sufficient resources available or when it exceeds their ability to cope [11, 23]. By expanding support services in school and among colleagues, the number of resources each teacher has available grows, and a challenge appraisal becomes more likely. These effects, however, were not examined in the present study and require further empirical investigation.

This study offers valuable insights into the field of teachers in vocational education and training, where research is still limited. In this study, we considered mental health-related concepts, teachers’ well-being, job satisfaction, risk of burnout, and experience of stress as outcomes. However, it is also important to analyze how the mental health of teachers in vocational education affects their quality of teaching, dropout rates, and the quality of the school. These measures have a more profound impact not only on the schools themselves but also on the economy and the educational system as a whole. Our research focused on the role of resilience and the personalities of VET teachers in the appraisal process. This, however, is only a limited list of possible antecedents, and the investigation of other influencing factors on the appraisal process requires further analysis. For example, gender, working hours, and working conditions might be interesting aspects to consider.

Finally, as mentioned above, the time of data collection for this study is a key limitation for the generalizability of the data. Therefore, a second study should be carried out with more space from the COVID-19 pandemic to rule out the effects of this pandemic and its associated restrictions.

## Conclusion

This study investigated how teachers in vocational education in Germany appraise job demands and how this appraisal affects their mental health and work experience. The findings showed that job demands in the category of *task scope and complexity* were perceived as occurring most frequently. The category *teaching and interacting with learners* was perceived almost as frequently, however it was also seen as the most challenging. Overall, the values for threat appraisal were generally higher than those of challenge appraisal, indicating that teachers see their job demands rather as threatening than challenging. Furthermore, it was found that the way sample of teachers in vocational education appraise job demands can influences their well-being, job satisfaction, and risk of burnout. Challenge appraisals have been found to be associated with more favorable outcomes, such as higher job satisfaction and lower risk of burnout, whereas threat appraisals are linked to unfavorable outcomes, such as lower well-being. Finally, resilience and certain personality traits were found to influence cognitive appraisal. Resilient teachers were more likely to perceive job demands as challenging and less likely to perceive them as threatening. In conclusion, these findings underline the important role of understanding how teachers in vocational education experience and deal with their daily job demands. These are valuable insights and might be helpful in addressing prevalent problems in the teaching profession, such as high burnout rates [3, 5], teacher shortages [7, 8], and a high prevalence of symptoms of psychological disorders [2]. 

## Supplementary Information

Supplementary Material 1.

Supplementary Material 2: Appendix 1. Assessment of demanding aspects for vocational education teachers (ADA-VET) Questionnaire in English. Appendix 2. Standardized Factor Loadings for the Six-Factor Model of Job Demands. Appendix 3. Pearson correlations among the six categories of job demands for the perception, challenge appraisal, and threat appraisal. Appendix 4. WHO-5 Well-Being Questionnaire. Appendix 5. Burnout Assessment Tool Short Version (BAT-12). Appendix 6. 10 Item Big Five Inventory. Appendix 7. Brief Resilience Scale.

## Data Availability

The dataset generated and/or analyzed in this study are available from the corresponding author upon reasonable request.

## Abbreviations

VET: Vocational Education and Training
RQ1/2/3: Research Question 1/2/3
H1/2/3: Hypothesis 1/2/3
JD-R: Job Demands-Resources Model
AARL-BS: Project on working hours, workload, and resilience of vocational education teachers in Baden-Württemberg, Germany (in German: Arbeitszeit, Arbeitsbelastung und Resilienz von Lehrkräften an beruflichen Schulen in Baden-Württemberg)
ADA-VET: Assessment of demanding aspects for vocational education teachers
BLV BW e.V.: Vocational Education Teachers’ Association of Baden-Württemberg, Germany[Berufsschullehrerverband Baden-Württemberg]
BRS: Brief Resilience Scale
BAT: Burnout Assessment Tool
WHO: World Health Organization
WHO-5: Well-being questionnaire by the World Health Organization
BFI-10: Big Five Inventory with 10 items
O: Openness
C: Conscientiousness
E: Extraversion
A: Agreeableness
N: Neuroticism
