# Supplementary material for: Challenge and threat appraisal of job demands and their impact on mental health among vocational education teachers
Source: BMC Public Health. 2026 Oct 8;26:2890. doi: 10.1186/s12889-026-29796-7 (PMC13647851; doi:10.1186/s12889-026-29796-7)
Supplement: Supplementary file 1 [file 12889_2026_29796_MOESM1_ESM.pdf]

Survey (English language version)

1. The work of a teacher at vocational schools presents many demanding aspects. Below are some of these job demands listed. How often does each job demand occur in your everyday working life?

	never	Every few months	Every few weeks	Every few days	daily
Lack of social recognition or poor image of the teaching profession in general	o	o	o	o	o
Low appreciation of vocational schools in education / educational policy compared to other types of schools	o	o	o	o	o
High pressure to reform vocational schools (e.g., new curricula, digitalization)	o	o	o	o	o
Lack of opportunities to cooperate with colleagues / 'lone wolf' attitude	o	o	o	o	o
Conflict with individual colleagues	o	o	o	o	o
Generally bad working atmosphere at my school (e.g., envy among the staff, lack of a culture of trust)	o	o	o	o	o
Conflict with school management	o	o	o	o	o
Lack of appreciation by the school management	o	o	o	o	o
Unclear communication by the school management	o	o	o	o	o
High workload	o	o	o	o	o
Feeling the need to do many different tasks at the same time	o	o	o	o	o
High time pressure	o	o	o	o	o
Principle incompleteness of the work tasks (as a teacher you can always do more)	o	o	o	o	o
Lack of feedback on the success of my work	o	o	o	o	o
Heterogeneous performance prerequisites of the students	o	o	o	o	o
Low motivation among the students	o	o	o	o	o
Disciplinary problems among students (e.g., disruptions in class, tardiness)	o	o	o	o	o
Many teacher roles, some of which are difficult to reconcile (e.g., learning partner, educator, grade giver)	o	o	o	o	o
Disruptive, hectic work environment (e.g., high noise level, few breaks, lack of retreats for rest)	o	o	o	o	o

High uncertainty about legal issues (e.g., data protection, grading, acceptability of excuses)	o	o	o	o	o
Insufficient technical equipment (e.g., WLAN, availability of tablets).	o	o	o	o	o

2. For each job demand, please indicate to what extent you perceive it as (A) a challenge and (B) a threat.
   Note: A job demand can be either a challenge or a treat, or both at the same time. Therefore, please always answer for (A) and (B).

	A. This job demand is a challenge for me.				B. This job demand is a threat for me			
	disagree strongly	disagree a little	agree a little	agree strongly	disagree strongly	disagree a little	agree a little	agree strongly
Lack of social recognition or poor image of the teaching profession in general	o	o	o	o	o	o	o	o
Low appreciation of vocational schools in education / educational policy compared to other types of schools	o	o	o	o	o	o	o	o
High pressure to reform vocational schools (e.g., new curricula, digitalization)	o	o	o	o	o	o	o	o
Lack of opportunities to cooperate with colleagues / ‘lone wolf’ attitude	o	o	o	o	o	o	o	o
Conflict with individual colleagues	o	o	o	o	o	o	o	o
Generally bad working atmosphere at my school (e.g., envy among the staff, lack of a culture of trust)	o	o	o	o	o	o	o	o
Conflict with school management	o	o	o	o	o	o	o	o
Lack of appreciation by the school management	o	o	o	o	o	o	o	o
Unclear communication by the school management	o	o	o	o	o	o	o	o

Conflict with the training organizations	o	o	o	o	o	o	o	o
Conflict with the legal guardians	o	o	o	o	o	o	o	o
Conflict with other external institutions (e.g., chambers of commerce, social services, youth welfare)	o	o	o	o	o	o	o	o
High workload	o	o	o	o	o	o	o	o
Feeling the need to do many different tasks at the same time	o	o	o	o	o	o	o	o
High time pressure	o	o	o	o	o	o	o	o
Principle incompleteness of the work tasks (as a teacher you can always do more)	o	o	o	o	o	o	o	o
Lack of feedback on the success of my work	o	o	o	o	o	o	o	o
Heterogeneous performance prerequisites of the students	o	o	o	o	o	o	o	o
Low motivation among the students	o	o	o	o	o	o	o	o
Disciplinary problems among students (e.g., disruptions in class, tardiness)	o	o	o	o	o	o	o	o
Many teacher roles, some of which are difficult to reconcile (e.g., learning partner, educator, grade giver)	o	o	o	o	o	o	o	o
Disruptive, hectic work environment (e.g., high noise level, few breaks, lack of retreats for rest)	o	o	o	o	o	o	o	o
High uncertainty about legal issues (e.g., data protection, grading, acceptability of excuses)	o	o	o	o	o	o	o	o
Insufficient technical equipment (e.g., WLAN, availability of tablets).	o	o	o	o	o	o	o	o

3. The following statements relate to your well-being over the past two weeks. To what extent do these statements apply to you?

Over the last two weeks...	All the time	Most of the time	Slightly more than half the time	Slightly less than half the time	Some of the time	At no time
...I feel cheerful and in good spirits.	o	o	o	o	o	o
...I feel calm and relaxed.	o	o	o	o	o	o
...I feel active and vigorous.	o	o	o	o	o	o
...I wake up feeling fresh and rested	o	o	o	o	o	o
...my daily life is filled with things that interest me.	o	o	o	o	o	o

4. Considering everything that plays a role in your work as a teacher at vocational schools: How satisfied are you overall with your professional situation at the moment?

Very unsatisfied	Rather unsatisfied	Neither satisfied not unsatisfied	Rather satisfied	Very satisfied
o	o	o	o	o

5. Considering everything that plays a role in your work as a teacher at vocational schools: How often do you feel exposed to situations that you experience as stressful?

never	Every few months	Every few weeks	Every few days	Every day
o	o	o	o	o

6. When you think back over the last 12 months, how often did you experience the following feelings?

	Never	Rarely	Sometimes	Often	Always

I struggle to find any enthusiasm for my work.	o	o	o	o	o
At work, I may overreact unintentionally.	o	o	o	o	o
I make mistakes in my work because my mind is on other things.	o	o	o	o	o
I'm cynical about what my work means to others.	o	o	o	o	o
I do not recognize myself in the way I react emotionally at work.	o	o	o	o	o
At work, I feel mentally exhausted.	o	o	o	o	o
After a day at work, I find it hard to recover my energy.	o	o	o	o	o
At work, I feel physically exhausted.	o	o	o	o	o
I feel a strong aversion toward my job.	o	o	o	o	o
At work, I have trouble staying focused.	o	o	o	o	o
At work, I feel unable to control my emotions.	o	o	o	o	o
When I'm working, I have trouble concentrating.	o	o	o	o	o

7. If you were to assess yourself personally: To what extent do each of the following statements apply to you?

I see myself as someone who...	Disagree strongly	Disagree a little	Neither agree nor disagree	Agree a little	Agree strongly
...is reserved	o	o	o	o	o
...is generally trusting	o	o	o	o	o
...tends to be lazy	o	o	o	o	o
...is relaxed, handles stress well	o	o	o	o	o
...has few artistic interests	o	o	o	o	o
...is outgoing, sociable	o	o	o	o	o
...tends to find fault with others	o	o	o	o	o

...does a thorough job	o	o	o	o	o
...gets nervous easily	o	o	o	o	o
...has an active imagination	o	o	o	o	o

8. How much do the following statements about stress recovery apply to you?

	Disagree strongly	Disagree a little	Neither agree nor disagree	Agree a little	Agree strongly
I tend to bounce back quickly after hard times.	o	o	o	o	o
I have a hard time making it through stressful events.	o	o	o	o	o
It does not take me long to recover from a stressful event.	o	o	o	o	o
It is hard for me to snap back when something bad happens.	o	o	o	o	o
I usually come through difficult times with little trouble.	o	o	o	o	o
I tend to take a long time to get over setbacks in my life.	o	o	o	o	o

Demographic information

9. Are you currently in a training program to acquire your first teaching qualification at vocational schools (e.g., preparatory service or direct entry)?

- o Yes, during preparatory service (teaching internship; incl. lateral entry)
- o Yes, with direct entry
- o Yes, namely:
- o No

10. Are you a member of the school management team?

- o Yes, I am the principal
- o Yes, I am the vice principal
- o Yes, I am the department head

- No

11. What is your gender?

- Female
- Male
- Divers

12. When were you born? Please enter your year of birth.
13. Are you working full-time or part-time during the current school year?

- I am working full-time
- I am working part-time
  Please enter your percentage:
