# Supplementary material for: Challenge and threat appraisal of job demands and their impact on mental health among vocational education teachers
Source: BMC Public Health. 2026 Oct 8;26:2890. doi: 10.1186/s12889-026-29796-7 (PMC13647851; doi:10.1186/s12889-026-29796-7)
Supplement: Supplementary file 2 [file 12889_2026_29796_MOESM2_ESM.odt]

Appendix 1. Assessment of demanding aspects for vocational education teachers (ADA-VET) Questionnaire in English.
Item-Nr.
Item
Category
1
Lack of social recognition or poor image of the teaching profession in general
Job demands in the context of the social and educational environment (1)
2
Low appreciation of vocational schools in education/educational policy compared to other types of schools
3
High pressure to reform vocational schools (e.g., new curricula, digitalization)
4
Lack of opportunities to cooperate with colleagues/“lone wolf” attitude
Social job demands among the colleagues/teaching staff (2)
5
Conflict with individual colleagues
6
Generally bad working atmosphere at my school (e.g., envy among the staff, lack of a culture of trust)
7
Conflict with school management
Social job demands with the school management (3)
8
Lack of appreciation by the school management
9
Unclear communication by the school management
10
High workload
Job demands in the context of task scope and complexity (4)
11
Feeling the need to do many different things/tasks at the same time
12
High time pressure
13
Principle incompleteness of the work tasks (as a teacher you can always do more)
14
Lack of feedback on the success of my work
15
Heterogeneous performance prerequisites of the learners
Job demands in the context of teaching or interacting with learners (5)
16
Low motivation among the students
17
Disciplinary problems among students (e.g., disruptions in class, tardiness)
18
Many teacher roles, some of which are difficult to reconcile (e.g., learning partner, educator, grade giver)
19
Disruptive, hectic work environment (e.g., high noise level, few breaks, lack of retreats for rest)
Job demands in the context of school frameworks (6)
20
High uncertainty about legal issues (e.g., data protection, grading, acceptability of excuses)
21
Insufficient technical equipment (e.g., WLAN, availability of tablets)

Appendix 2. Standardized Factor Loadings for the Six-Factor Model of Job Demands.
Factor
Item-Nr.
Standardized factor loadings
1 – Job demands in the context of the social and educational environment
1
.641
2
.635
3
.521
2 – Social job demands among the colleagues / teaching staff
4
.509
5
.679
6
.845
3 – Social job demands with the school management
7
.817
8
.875
9
.823
4 – Job demands in the context of task scope and complexity
10
.746
11
.852
12
.870
13
.701
14
.430
5 – Job demands in the context of teaching or interacting with learners
15
.551
16
.762
17
.759
18
.644
6 – Job demands in the context of school framework
19
.723
20
.637
21
.319
Note. Factor loadings are standardized estimates from the confirmatory factor analysis of the proposed six-factor model for the job demands.

Appendix 3. Pearson correlations among the six categories of job demands for the perception, challenge appraisal, and threat appraisal.
Job demand category
1
2
3
4
5
6
Perception
Category 1
1
.328
.234
.410
.243
.304
Category 2
-
1
.486
.319
.209
.327
Category 3
-
-
1
.222
.132
.279
Category 4
-
-
-
1
.433
.460
Category 5
-
-
-
-
1
.500
Category 6
-
-
-
-
-
1
Challenge appraisal
Category 1
1
.400
.436
.527
.426
.416
Category 2
-
1
.589
.467
.332
.488
Category 3
-
-
1
.510
.341
.533
Category 4
-
-
-
1
.487
.541
Category 5
-
-
-
-
1
.426
Category 6
-
-
-
-
-
1
Threat appraisal
Category 1
1
.326
.240
.451
.346
.318
Category 2
-
1
.425
.282
.242
.303
Category 3
-
-
1
.200
.182
.286
Category 4
-
-
-
1
.437
.403
Category 5
-
-
-
-
1
.365
Category 6
-
-
-
-
-
1


Appendix 4. WHO-5 Well-Being Questionnaire.
Item (English)
WHO [45]
Item (German)
Brähler et al. [46]
Over the last two weeks 
In den letzten zwei Wochen
I feel cheerful and in good spirits.
war ich froh und guter Laune.
I feel calm and relaxed.
habe ich mich ruhig und entspannt gefühlt.
I feel active and vigorous.
habe ich mich energisch und aktiv gefühlt.
I wake up feeling fresh and rested.
habe ich mich beim Aufwachen frisch und ausgeruht gefühlt.
My daily life is filled with things that interest me.
war mein Alltag voller Dinge, die mich interessieren.

Appendix 5. Burnout Assessment Tool Short Version (BAT-12).
Item (English)
Schaufeli et al. [48]
Item (German)
Glaser & Seubert [49]
I struggle to find any enthusiasm for my work.
Ich kann keine Begeisterung für meine Arbeit aufbringen.
At work I may overreact unintentionally.
Bei meiner Arbeit kommt es vor, dass ich emotional überreagiere, ohne es zu wollen.
I make mistakes in my work because my mind is on other things.
Ich mache Fehler bei meiner Arbeit, weil ich in Gedanken bei anderen Dingen bin.
I’m cynical about what my work means to others.
Ich bezweifle, dass meine Arbeit anderen überhaupt etwas bedeutet.
I do not recognize myself in the way I react emotionally at work.
Ich erkenne mich selbst nicht wieder in der Art, wie ich bei der Arbeit emotional reagiere.
At work, I feel mentally exhausted.
Durch meine Arbeit fühle ich mich psychisch erschöpft.
After a day at work, I find it hard to recover my energy.
Nach einem Arbeitsalltag fällt es mir schwer, mich zu erholen.
At work, I feel physically exhausted.
Durch meine Arbeit fühle ich mich körperlich erschöpft.
I feel a strong aversion toward my job.
Ich fühle eine starke Abneigung gegen meine Arbeit.
At work, I have trouble staying focused.
Bei der Arbeit habe ich Mühe, aufmerksam zu bleiben.
At work, I feel unable to control my emotions.
Bei meiner Arbeit habe ich das Gefühl, keine Kontrolle über meine Emotionen zu haben.
When I’m working, I have trouble concentrating.
Bei der Arbeit habe ich Mühe, mich zu konzentrieren.

Appendix 6. 10 Item Big Five Inventory.
Item (English)
Rammstedt & John [52]
Item (German)
Rammstedt & John [52]
I see myself as someone who is reserved.*
Ich bin eher zurückhaltend, reserviert.*
I see myself as someone who is generally trusting.
Ich schenke anderen leicht Vertrauen, glaube an das Gute im Menschen.
I see myself as someone who tends to be lazy.*
Ich bin bequem, neige zur Faulheit.*
I see myself as someone who is relaxed, handles stress well.*
Ich bin entspannt, lasse mich durch Stress nicht aus der Ruhe bringen.*
I see myself as someone who has few artistic interests.*
Ich habe nur wenig künstlerisches Interesse.*
I see myself as someone who is outgoing, sociable.
Ich gehe aus mir heraus, bin gesellig.
I see myself as someone who tends to find fault with others.*
Ich neige dazu, andere zu kritisieren.*
I see myself as someone who does a thorough job.
Ich erledige meine Aufgaben gründlich.
I see myself as someone who gets nervous easily.
Ich werde leicht nervös und unsicher.
I see myself as someone who has an active imagination.
Ich habe eine aktive Vorstellungskraft, bin fantasievoll.
* Item is reversed-scored.
Appendix 7. Brief Resilience Scale.
Item (English)
Smith et al. [53]
Item (German)
Chmitorz et al. [54]
I tend to bounce back quickly after hard times.
Ich neige dazu, mich nach schwierigen Zeiten schnell zu erholen.
I have a hard time making it through stressful events. *
Es fällt mir schwer, stressige Situationen durchzustehen. *
It does not take me long to recover from a stressful event.
Ich brauche nicht viel Zeit, um mich von einem stressigen Ereignis zu erholen.
It is hard for me to snap back when something bad happens. *
Es fällt mir schwer zur Normalität zurückzukehren, wenn etwas Schlimmes passiert ist. *
I usually come through difficult times with little trouble.
Normalerweise überstehe ich schwierige Zeiten ohne größere Probleme.
I tend to take a long time to get over setbacks in my life. *
Ich brauche tendenziell lange, um über Rückschläge in meinem Leben hinwegzukommen. *
* Inverted item.
